# Natural history and outcome of nonketotic hyperglycinemia in China

**DOI:** 10.3389/fneur.2024.1440883

**Published:** 2024-08-14

**Authors:** Zhizi Zhou, Yanna Cai, Xiuzhen Li, Zongcai Liu, Minzhi Peng, Yunting Lin, Xiaojian Mao, Chunhua Zeng, Li Liu, Wen Zhang

**Affiliations:** Department of Genetics and Endocrinology, Guangzhou Women and Children’s Medical Center, Guangzhou Medical University, Guangzhou, Guangdong, China

**Keywords:** nonketotic hyperglycinemia, GLDC, AMT, glycine encephalopathy, genotype

## Abstract

**Introduction:**

Nonketotic hyperglycinemia (NKH) is a rare, life-threatening genetic disorder. The patients usually show heterogeneous and nonspecific symptoms, resulting in diagnosis challenges using conventional approaches. Here, the clinical presentation and genetic features of 20 Chinese patients were examined and reported in order to clarify the natural history and prognosis of NKH in China.

**Methods:**

The Human Gene Mutation Database and literature regarding NKH in China were reviewed. Age of onset, clinical characteristics, genetic analysis, cranial magnetic resonance imaging (MRI) and electroencephalography (EEG) examinations, and outcome of the patients were analyzed. Natural history experiences and follow-up assays for five patients who were followed in our center were described.

**Results:**

Among all 20 NKH patients, 17 (85%) had the neonatal type and 3 (15%) had the infantile type, no late-onset cases were detected. Patients showed up for admission with a history of seizures (15/20), lethargy (14/20), hypotonia (11/20), apnea (9/20), and feeble sobbing (4/20). Brain MRI findings included abnormal signals in the internal capsule, cerebellum, or brainstem (6/14), dysplasia of the corpus callosum (5/14), and white matter abnormalities (3/14). EEG evaluations showed anomalies such as burst suppression (4/8) and hypsarrhythmia and/or epileptic activity (6/8). Median values of cerebrospinal fluid (CSF) glycine levels, plasma glycine levels and CSF/plasma glycine ratios were135.2 (range, 6.3–546.3) μmol/L, 998.2 (range,75–3,084) μmol/L, 0.16 (range, 0.03–0.60) respectively. Genetic analyses revealed four new variations and *GLDC*, *AMT* gene abnormalities in 13 (65%), 7 (35%) case, respectively. Prognosis information was available for 18 cases: nine patients died, eight in the neonatal period. Among the nine survivors, varying developmental disorders were observed.

**Discussion:**

Different disease processes and outcomes were found in Chinese NKH patients, according to this study. The initial clinical presentations, CSF glycine levels and CSF to plasma glycine ratios do not reliably predict prognosis, while MRI and EEG abnormalities may indicate a poor outlook. NKH diagnosis should be considered for neonates presenting specific symptoms. The present survey provides clinical data that support the development of a standardized protocol for diagnosing and treating NKH in China.

## 1 Introduction

Autosomal recessive nonketotic hyperglycinemia (NKH; MIM#605899) is caused by a biochemical defect in the glycine cleavage system (GCS). NKH is distributed worldwide, with an estimated incidence of 1/76,000 (1). The highest incidence has been reported in northern Finland, with a prevalence of 1:12,000 (2). The incidence in mainland China remains unknown, and 15 cases have been reported to date. Its incidence in Taiwan is 7.2/1,000,000 (3).

NKH manifests as a variety of nonspecific symptoms, making it difficult to diagnose using traditional detection techniques. The identification and diagnosis of ailments are further complicated by the lack of uniform reference values throughout laboratories. Based on clinical manifestations and outcomes, NKH can be divided into severe and attenuated forms (4). According to the age of onset, NKH can be categorized as neonatal (classical; 0–4 weeks), infantile (5 weeks–2 years), and late-onset (>2 years) (5). The prognosis for typical cases is devastate, with the majority of patients succumbing in early infancy. Survivors often experience severe developmental delays and uncontrollable seizures.

NKH is extremely rare and may vary among ethnicities. So far, prospective natural history studies of NKH in China are lacking. To expand the knowledge and reported information regarding clinical experiences related to NKH, the present study describes the natural history and follow-up assays for five patients (cases 16–20) who were followed in our center.

## 2 Case reports

### 2.1 Case 1

A 6-month-old girl was brought to the emergency department because of repetitive lethargy, and polypnea. She was 5th child of a healthy nonconsanguineous couple. The first was a healthy girl, while the other three were terminated pregnancy due to fetal cessation of development in early pregnancy. She was born at 40^+5^ gestation weeks by cesarean section due to acute fetal distress. The Apgar scores were 8 at 1 min and 9 at 5 min.

At 2-day, she was admitted in the NICU because of lethargy, hypotonia and pneumonia. Respiratory acidosis was detected on arterial blood gas analysis. She developed first episode of fever-provoked generalized tonic seizures at 6-months old. Laboratory evaluation performed at 6-months old detected levels of CSF/plasma glycine ratio of 0.1. Plasma amino acid and CSF glycine levels were elevated to 520.8 and 52.9 μmol/L, respectively. Biochemical investigation, serum lactate and ammonia, plasma acylcarnitine and urine organic acid profiles were normal. Etiological examination was negative. Background of EEG recorded at 6 months showed increased slow activity, mainly medium and high amplitude slow waves. Brain MRI showed bilateral widening of the extracerebral space and increased signal intensity in the globus pallidus. Genetic analysis showed compound heterozygous mutations in the *GLDC* gene (reference genome hg 19, NM_000170.2) c.2405C > T (p.A802V) and c.2639A > G(p.D880G), which were inherited from the mother and the father, respectively. Sodium benzoate and levocarnitine were prescribed to control convulsions and improve alertness. Protein-restricted diet (1.5 g/kg/day) was also tried to the baby.

The child’s convulsions improved by the age of 10 months after treatment, but she displayed increased irritability. At 3 months, the GESELL score was 47 points. By 5 months, she could lift her head, and by 1 year and 11 months, she was able to assist in standing. By 3-years old, she was seizures-free and could walk unaided with an abnormal gait. At 4-years old, she could consciously call “Mom and Dad,” understand simple instructions and learn to grasp. Limbs shake during fever, hypotonia, choreiform movement disorders, hyperextension of knees, overriding toes and bilateral clubfeet were observed.

### 2.2 Case 2

A 2-months-old baby boy was referred due to recurrent seizures (4–6 times per day). He was the second child of a healthy nonconsanguineous couple, and the pregnancy and delivery were uneventful. The infant was born at term via cesarean section with an Apgar score of 10/10.

Following delivery, he was admitted to the NICU due to weak crying, hypotonia and feeding difficulties. Twelve hours later, the infant experienced convulsions and respiratory distress, necessitating intubation and mechanical ventilation. Arterial blood gas analysis revealed respiratory acidosis.

On 14th day, plasma and CSF analysis showed elevated levels of glycine at 532.3 and 93.4 μmol/L, respectively. The CSF/plasma glycine ratio was 0.18. Cranial ultrasound revealed ventriculomegaly, while brain MRI demonstrated periventricular white matter hyperintensities and a thin corpus callosum. The EEG findings indicated a pattern of hypsarrhythmia or burst suppression. A clinical exome study revealed compound heterozygous variants of the *AMT* gene (reference genome hg 19, NM_000481.3) c.878-1G > A and c.781A > G (p.R261G).

The infant received levetiracetam as antiepileptic therapy, and was prescribed sodium benzoate, as well as cofactors such as levocarnitine and folinic acid, to improve alertness. Despite treatment, the patient exhibited poor seizure control and was lost to follow-up after discharge.

### 2.3 Case 3

A 5-day-old male infant was referred to NICU with decreased activity and hypopnea. He was the second child of nonconsanguineous parents, who had a healthy daughter. During the second trimester, fetal agenesis of the corpus callosum and nuchal translucency were identified via ultrasound examination. The infant was born at 39^+5^ gestation weeks through normal vaginal delivery, with an Apgar score of 10/10. Following delivery, the infant exhibited weak crying, decreased activity, and feeding difficulties. At the 3rd day, the infant’s condition deteriorated, leading to expiratory dyspnea, necessitating intubation and mechanical ventilation.

A cranial MRI performed on the 7th day revealed agenesis of the corpus callosum. An EEG during the same period showed a discontinuous pattern of background activity without sleep–wake cycles, with six sharp waves observed within 3 h, and concurrent unilateral limb twitching. Seizures were reduced after Lumina treatment.

At the 10th day, amino acid and acyl carnitine profiles were obtained, leading to a diagnosis based on markedly elevated glycine levels in plasma and CSF, measuring 998.2 and 546.3 μmol/L, respectively. The CSF/plasma glycine ratio was notably high at 0.55. A homozygous mutation p.D276H in the *AMT* gene was identified, with both parents being heterozygous for his mutation.

Dextromethorphan and sodium benzoate were administered to reduce plasma glycine concentration. By one and a half years of age, the infant continued to undergo 2 to 4 convulsions per day and exhibited developmental delays, including an inability to roll over and speak.

### 2.4 Case 4

A 3-months-old male infant was brought to the rare disease clinic by her parents because of intractable seizures. He was the second child of healthy nonconsanguineous parents who also had a healthy daughter. The infant was born at term through normal vaginal delivery.

On 3rd day, the child was hospitalized in the NICU because of lethargy, myoclonic jerks and seizures. Initial amino acid profiling at the local hospital revealed dramatically elevated levels of plasma glycine to 1119.3 μmol/L. The follow-up amino acid profiling at our center, performed at 27-days old, showed glycine levels at 490 μmol/L. Urine organic acid analysis did not yield any abnormalities. Unfortunately, consent for CSF glycine measurement could not be obtained from the parents. Based on the significantly elevated glycine levels, nonketotic hyperglycinemia was suspected. Whole-exome sequencing (WES) identified compound heterozygous mutations, c.125A > C (p.H42P) and c.826G > C (p.D276H), in *AMT* gene. Cranial MRI performed at 3 years old revealed extracerebral space and bilateral ventricles.

By the age of 3-year and 2-month, the child experienced minor epilepsy 4–5 times per day without receiving drug treatment. He exhibited significant developmental delays, including an inability to raise his head and speak. He was exclusively fed a semiliquid diet.

### 2.5 Case 5

A 5-year-old boy was brought to the rare disease clinic due to the developmental delay. He was the first child of nonconsanguineous parents, and his pregnancy, labor, and delivery were uneventful. He was born at 41 weeks gestation through natural labor, and he did not experience any neonatal complication.

Concerns arose when his parents noticed developmental delays, because he was unable to raise his head at 3 months. He experienced his first convulsion during a febrile illness at 6-month, which ceased after he grew to 3-year-old. At 3-years old, he began walking but exhibited truncal instability and an ataxic gait. He started pronouncing “Baba, Mama” at 4-years old.

Routine laboratory findings, including blood, urine, and cerebrospinal fluid were normal. Arterial blood gas analysis and serum ammonia levels did not indicate any abnormalities. His plasma glycine levels were measured at 563.9 μmol/L, and unfortunately, CSF glycine measurement could not be performed. EEG and brain MRI performed at 5-years old did not show any abnormality. NKH was confirmed through WES, which identified compound heterozygous mutations in the *AMT* gene (c.550 + 4A > G and c.664C > T). The child received cofactor administration (levocarnitine, vitamins and folinic acid) along with a protein-restricted diet (1.5 g/kg/day).

He achieved independent walking at the age of 3 and began speaking at 5-year-old. From age of 4–7, he continued to reach developmental milestones and was enrolled in special education classes. At 7-year-old, he had a vocabulary of 5 words. While his gross motor development was slightly delayed compared to his peers, he struggled with fine motor movements.

## 3 Materials and methods

### 3.1 Subjects

The clinical suspicion criteria of NKH are based on the patient’s clinical presentation, detailed neurological examinations, seizure semiology, EEG results, and neuroimaging findings. Common pediatric problems such as infection, trauma, hypoxia have been ruled out. A subset of these patients underwent testing for plasma and/or cerebrospinal fluid glycine levels and the CSF/plasma glycine ratio. Samples from individuals suspected of NKH were sent to the DNA laboratory for diagnostic confirmation. The diagnosis was confirmed through direct sequencing of all exons and intron-exon boundaries of GLDC, AMT and GCSH. WES was performed in select patients, following the manufacturer’s protocol diligently. Bioinformatic analysis of the mutations was carried out using PROVEAN, SIFT, Mutation Taster, PolyPhen-2, and FATHMM to predict pathogenicity. Numerical variables were presented as median (min-max) based on the distribution normality.

From 2018 to 2023, five individuals from 5 different families in our hospitals were confirmed diagnosis of NKH. The study underwent review and approval by the ethics committees at our institution. Written informed consent for participation was obtained from all adult participants or guardians of minors or incapacitated subjects.

#### 3.1.1 Literature review

We conducted searches in the China National Knowledge Infrastructure and WanFang digital database as well as PubMed database from 2017 to 2023 using the terms: “nonketotic hyperglycinemia,” “glycine encephalopathy,” “GLDC,” “AMT” and “glycine cleavage enzyme system.” Additionally, we consulted the HGMD Professional database for the same period. Data extraction encompassed pregnancy and perinatal details, clinical data, genetic analysis, information on supplementary tests, neuroimaging results, as well as outcomes.

## 4 Results

### 4.1 Clinical findings

A total of 20 cases, comprising 15 NKH patients from 12 distinct families reported in mainland China between 2017 and 2023, have been included in this study. Detailed information is depicted in Table 1 (6–16). The parents of all patients were healthy and non-consanguineous. All patients were born at term and experienced uneventful deliveries. Cases 3 and 4, as well as cases 7, 8, and 9, involved siblings. Among the 17 infants for whom birth information was available, all had a normal birth weight.

**Table 1:** Clinical and biochemical findings of Chinese patients with NKH.

Patient	Birth weight(g)	Age of onset	Clinical features	Plasma Gly (μmol/L)	CSF Gly (μmol/L)	CSF/plasma gly ratio	Brain MRI	EEG	Treatment	Outcome	Ref
Case 1	3,150	1 d	Poor feeding, decreased activity, lethargy, hypotonia, intractable seizures (after treatment, seizures dropped from 10 times daily to once or twice) developmental delay	947.8 (Nr:115 ∼ 600)	226.4 (Nr:3 ∼ 20)	0.24 (Nr:<0.02)	Dysplasia of corpus callosum	NA	Sodium benzoate, pyridoxine, Dextromethorphan Dietary restriction	Alive at 1 year 7 months: improved deep tendon reflexes and muscular hypotonia; but still poor feeding and intellectual disability	6
Case 2	3,120	11 Hours	Seizures (after treatment, epilepsy is reduced by 50–60%) metabolic Encephalopathy	Normal	NA	NA	Dysplasia of corpus callosum; High signal of bilateral inner capsule, midbrain, dorsal pons	Burst suppression	Adreno-corticotropic-hormone, Topiramate, and Dextromethorphan	Died at 4 m	7
Case 3	NA	9 Months	Seizures (over 10 times a day)	75 (Nr:0 ∼ 276)	45.3 (Nr:1.6 ∼ 19.5)	0.60 (Nr:<0.08)	Normal	Widespread epileptoid discharges	NA	Seizure (once every few months), severe bilateral spastic paralysis and developmental retardation (unable to sit independently or speak at 6 years and 8 months old)	8
Case 4	NA	2 Years	Ataxia, chorea, and behavioral abnormality	Normal	36.7 (Nr:1.6 ∼ 19.5)	0.13 (Nr:<0.02)	Normal	Generalized, intermittent, rhythmic activity	NA	Alive at 3 year 5 months: only able to say “dad” and “mom,” comprehend simple phrases, ataxia, chorea, walked unstable, behavioral problem	8
Case 5	3,200	After Delivery	Weak crying, lethargy, and seizures	1,304 (Nr:130 ∼ 650)	NA	NA	Decreased white matter density in the white matter of brain	Discontinuous patterns mainly, alternating patterns slightly	Antibiotics, mechanical ventilation and nutritional support	Died after 4 d	9
Case 6	NA	5 d	Poor feeding, lethargy, and limb tremor	1,409 (Nr:125 ∼ 750)	NA	NA	NA	NA	Cardiopulmonary resuscitation and trachea intubation and mechanicalventilation	Died at 6 d	10
Case 7	Normal	2nd	Hypotonia, lethargy, apnea, and seizures	NA	NA	NA	NA	NA	NA	Died at 11 d	11
Case 8	Normal	2nd	Lethargy, apnea, hiccup, and seizures	1,588 (Nr:232 ∼ 740)	260.2 (Nr:2.2 ∼ 14.2)	0.16 (Nr:<0.08)	NA	NA	NA	Died at 13 d	11
Case 9	Normal	3nd	Hypotonia, lethargy, hiccup and seizures	1,038 (Nr:232 ∼ 740)	157.2 (Nr:2.2 ∼ 14.2)	0.15(Nr:<0.08)	Extensive white matter diffusion restriction extending to the subcortical white matter	NA	Sodium benzoate, Dextromethorphan	Alive at 7 m: severe intellectual disability, frequent seizures	11
Case 10	3,600	2nd	Poor feeding, hypotonia, lethargy, apnea, and seizures	NA	NA	NA	Abnormal signals of anterior and posterior limbs of bilateral internal capsules, part of thalamus, brainstem, bilateral hemioval center and cerebellar dentate nucleus	Multifocal sharp waves and sharp slow waves	Phenobarbital and mechanical ventilation	Died at 7 d	12
Case 11	3,300	2nd	Poor feeding, hypotonia, lethargy, apnea, and seizures	NA	NA	NA	High signal of bilateral hemioval center, bilateral inner capsule anterior and posterior limbs, hippocampus, cerebellum and brainstem	NA	Phenobarbital and mechanical ventilation	Died 2 days after discharge	12
Case 12	2,600	After Delivery	Poor feeding, hypotonia, lethargy, and weak crying	942 (Nr:100 ∼ 500)	113.1	0.12(Nr:<0.08)	The thin and short of corpus callosum	NA	Antibiotics, mechanical ventilation, nutritional support and levocarnitine	Died at 13 d	13
Case 13	3,750	After Delivery	Poor feeding, hypotonia, apnea, hiccup, and seizures (3 ~ 5 times a day)	850(Nr:130 ∼ 500)	NA	NA	Abnormal signals of anterior and posterior limbs of bilateral internal capsules, cerebellar dentate nucleus	Burst suppression	Levetiracetam and clonazepam	Alive at 2 months, frequent seizures and muscular hypotonia	14
Case 14	3,785	2nd	Hypotonia, lethargy, hiccup, and seizures	1,470 (Nr:131 ∼ 368)	215 (Nr:3 ∼ 10)	0.15(Nr:<0.02)	High signal of posterior limbs of bilateral internal capsules, brainstem	Burst suppression	Sodium benzoate, folic acid and mechanical ventilation	Died at 20 d	15
Case 15	2,970	2nd	Lethargy, hypotonia, and apnea	3,084 (Nr:125 ∼ 450)	NA	NA	Symmetrical cytotoxic edema in the hind limbs of bilateral internal capsule, the midbrain, and the pons	NA	Mechanical ventilation	NA	16
Case 16	2,750	2nd	Lethargy, hypotonia, apnea, seizures	520.8 (Nr:119.0 ∼ 314.0)	52.9 (Nr:3.7 ∼ 7.6)	0.10 (Nr < 0.04)	Widening of the extracerebral space and increased signal intensity in the bilateral globus pallidus	Activity, mainly medium and high amplitude slow waves	Luminal, sodium benzoate, levocarnitine, dietary restriction of natural protein intake	Alive at 4 years, seizures-free, walked alone, choreiform movement disorders	Present study
Case 17	2,800	After Delivery	Poor feeding, weak crying, hypotonia, apnea, seizures	532.3 (Nr:119.0 ∼ 314.0)	93.4 (Nr:3.7 ∼ 7.6)	0.18 (Nr < 0.04)	Periventricular white matter hyperintensities and thin corpus callosum	Hypsarrhythmia or burst suppression	Luminal, levetiracetam, folinic acid and mechanical ventilation	Lost to follow-up	Present study
Case18	3,000	After Delivery	Lethargy, poor feeding, weak crying, and apnea	998.2 (Nr:119.0 ∼ 314.0)	546.3 (Nr:3.7 ∼ 7.6)	0.55 (Nr < 0.04)	Dysplasia of corpus callosum	A discontinuous pattern of background activity, sharp waves	Dextromethorphan, sodium benzoate and mechanical ventilation.	Alive at 18 months, seizures, severe developmental delay	Present study
Case 19	4,000	After Delivery	Lethargy, myoclonic, and seizures	1119.3 (Nr:119.0 ∼ 314.0)	NA	NA	Extracerebral space and bilateral ventricles	NA	Dietary restriction	Alive at 3 year 2 months, seizures, choreiform movement disorders	Present study
Case 20	3,200	3 months	Seizures, ataxic gait, and developmental delay	563.9 (Nr:119.0 ∼ 314.0)	NA	NA	Normal	Normal	Levocarnitine, vitamin and folinic acid	Alive at 7 years, walked alone, language retardation	Present study

NKH, nonketotic hyperglycinemia; Gly, glycine; CSF, cerebrospinal fluid; MRI, magnetic resonance imaging; EEG, electroencephalography; Nr, normal range; NA, not available.

**Figure 1:**
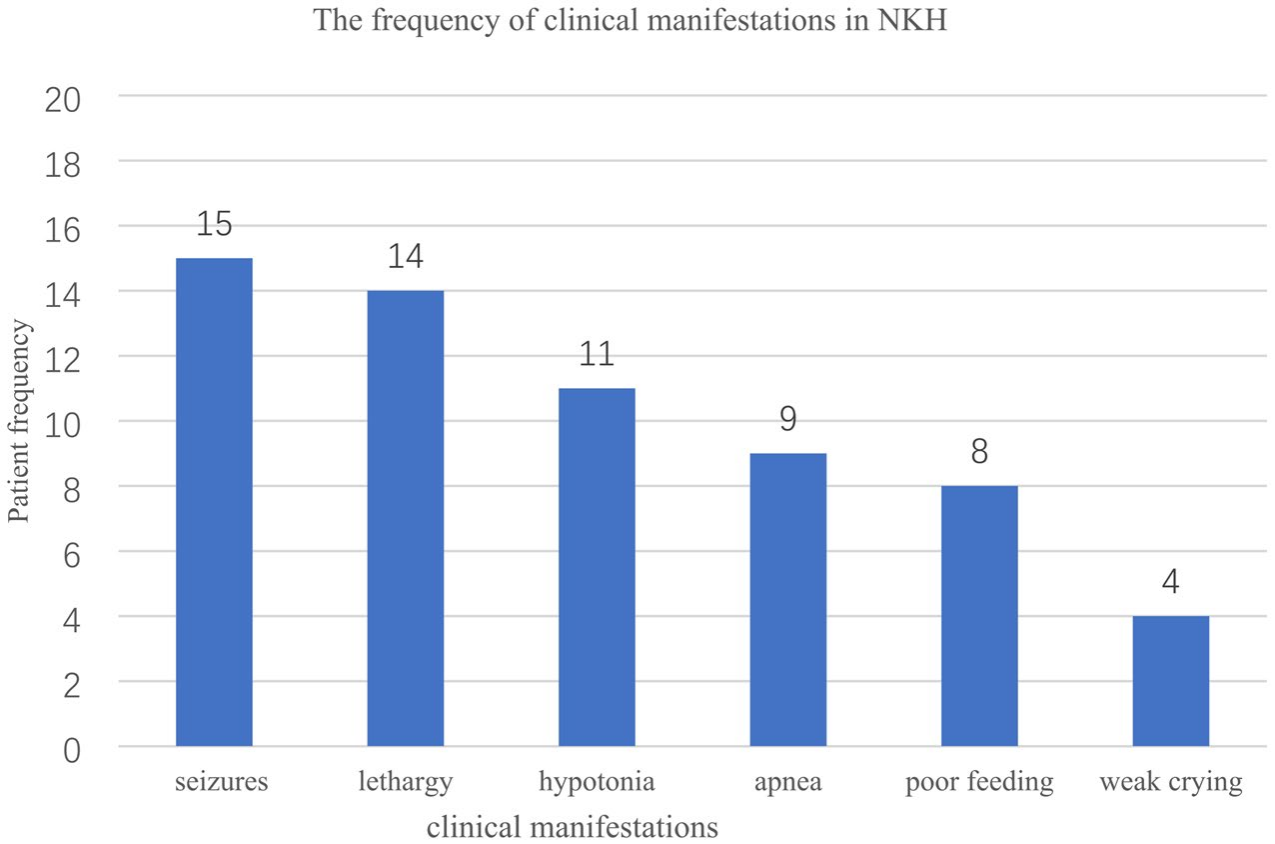
The frequency of clinical manifestations in 20 Chinese patients with NKH.

The male-to-female ratio was 15:5. Seventeen out of twenty (85.0%) patients had the neonatal type, while three ones (15.0%) had the infantile type, with no late-onset cases identified. Seventeen out of twenty (80.0%) patients exhibited the severe form, while three ones (15.0%) (Cases 4, 16, and 20, comprising one male and two females) reached the walking and speaking milestone. Symptoms at the time of hospital admission included seizures (15/20), lethargy (14/20), hypotonia (11/20), apnea (9/20), poor feeding (8/20), and weak crying (4/20) (Figure 1). Nine patients experienced respiratory distress and/or apnea requiring artificial ventilation, all of which were neonatal onset. Prognosis was provided for 18 cases, with nine patients succumbing, eight during the neonatal period. Nine patients were surveyed, and survivors exhibited different degrees of developmental disorders.

Plasma glycine concentration was measured in 17 patients, with significant elevation observed in 15 patients and normal levels detected in 2 patients, yielding a median value of 998.2 (range, 75–3,084) μmol/L. Cerebrospinal fluid glycine levels and CSF/plasma glycine ratios were measured in 10 cases. Elevated levels were observed in all cases, with a median value of 135.2 (range, 6.3–546.3) μmol/L, and 0.16 (range, 0.1–0.60) respectively.

Cranial MRI scans were conducted in 17 patients, with 14 showing abnormalities. Brain MRI abnormalities included abnormal signals in the internal capsule, cerebellum, or brainstem (6/14), dysplasia of the corpus callosum (5/14), and white matter abnormalities (3/14). EEG evaluations were performed in 10 cases, with eight cases showing abnormalities, including burst suppression (4/8) and hypsarrhythmia and/or epileptic activity (6/8).

### 4.2 Genetic findings

Thirteen cases (65%) exhibited genetic abnormalities in the *GLDC* gene, seven cases (35%) in the *AMT* gene, no patient had a mutation identified in *GCSH* gene among the 20 Chinese children with NKH (Table 2) (1, 6–23). All mutations were determined to be inherited.

**Table 2:** Molecular genetic findings of Chinese patients with NKH.

Patient	Gender	Gene	Exon/Intron	Nucleotide change	Amino acid change	Type of mutation	Reference(s)
Case 1	M	GLDC	Exon 21	c.2516A > G	p.Y839C	Missense	(7)
			Intron 21	c.2457 + 2 T > A		Splicing	(6)
Case 2	M	GLDC	Exon 15	c.1786 > T	p.R596X	Nonsense	(7)
			Exon 4–15	Exon 4–15 del		Deletion	(7)
Case 3	M	GLDC	Exon 25	c.3006C > G	p.C1002W	Missense	(8)
			Exon 9	c.1256C > G	p.S419X	Nonsense	(8)
Case 4	FM	GLDC	Exon 25	c.3006C > G	p.C1002W	Missense	(8)
			Exon 9	c.1256C > G	p.S419X	Nonsense	(8)
Case 5	M	GLDC	Exon 18	c.2198 > T	p.A733V	Missense	(17)
			Exon 18	c.2198 > T	p.A733V	Missense	(17)
Case 6	M	GLDC	Exon 13	c.1607G > A	p.R536Q	Missense	(18)
				9p24.3p22.3 del		Deletion	(10)
Case 7	M	GLDC	Exon 23	c.2680A > G	p.T894A	Missense	(11)
			Exon 3	Exon 3 del		Deletion	(11)
Case 8	M	GLDC	Exon 23	c.2680A > G	p.T894A	Missense	(11)
			Exon 3	Exon 3 del		Deletion	(11)
Case 9	M	GLDC	Exon 23	c.2680A > G	p.T894A	Missense	(11)
			Exon 3	Exon 3 del		Deletion	(11)
Case 10	M	GLDC	Exon 11	c. 1,470 T > A	p.C490X	Nonsense	(12)
			Exon 2	c.319_320delA	p.M107Wfs*123	Indel	(12)
Case 11	FM	AMT	Exon 8	c.992G > A	p.R331Q	Missense	(1)
			Exon 8	c.992G > A	p.R331Q	Missense	(1)
Case 12	FM	GLDC	Exon 9	c.1261 G > C	p.G421R	Missense	(13)
			Exon 3	c.450 > G	p.N150K	Missense	(13)
Case 13	M	GLDC	Exon 18	c.2182G > A	p.G728R	Missense	(19)
			Exon 3	c.395C > A	p.S132X	Nonsense	(14)
Case 14	M	AMT	Exon 6	c.664C > T	p.R222C	Missense	(18)
			Exon 7	c.793C > T	p.R265C	Missense	(20)
Case 15	FM	AMT	Exon 8	c.977delA	p.E326Gfs*12	Indel	(16)
			Exon 8	c.982_983insG	p.A328Gfs*22	Indel	(16)
Case 16	FM	GLDC	Exon 21	c.2405C > T	p.A802V	Missense	(21)
			Exon 22	c.2639A > G	p.D880G	Missense	Present study
Case 17	M	AMT	Intron 7	c.878-1G > A		Splicing	(22)
			Exon 7	c.781A > G	p.R261G	Missense	Present study
Case 18	M	AMT	Exon 7	c.826G > C	D276H	Missense	(23)
			Exon 7	c.826G > C	D276H	Missense	(23)
Case 19	M	AMT	Exon 2	c.125A > C	H42P	Missense	Present study
			Exon 7	c.826G > C	p.D276H	Missense	(23)
Case 20	M	AMT	Intron 5	c.550 + 4A > G		Splicing	Present study
			Exon 6	c.664C > T	p.R222C	Missense	(20)

NKH, nonketotic hyperglycinemia; F, female; M, male.

In this study, we identified 19 *GLDC* gene variants, including 10 missense mutations (52.6%), 1 splice site mutation (5.3%), 4 nonsense mutations (21.0%), 1 insertion/deletion (5.3%), and 3 copy number variations (CNVs) (15.8%). Notably, one mutation was novel (Table 2). In case 6, a 15.2 Mb deletion encompassing the 9p24.3p22.3 region, where the *GLDC* gene is located, was detected.

A total of 10 mutations (including 3 novel mutations) in the *AMT* gene were identified, including 6 missense mutations (60.0%), 2 splice site mutations (20.0%), and 2 insertions/deletions (20.0%).

In addition, we have identified four novel compound heterozygous variations, including c.2639A > G (p.D880G) in the *GLDC* gene; c.125A > C (H42P), c.781A > G (p.R261G) and c.550 + 4A > G in the *AMT* gene (Table 2).

## 5 Discussion

We present a comprehensive review of patients with NKH in China aiming to elucidate the clinical spectrum and outcomes of NKH. The data reveals a predominance of the infantile form of NKH in China, aligning well with the existing literature, with neonatal mortality observed in 47.1% (8/17) of severe cases. While previous literature (24) suggested gender differences in NKH prevalence, our study found more male patients with an equal distribution of mild cases between genders, highlighting uncertainties in gender-based incidence patterns in China. It remains unclear whether the incidence of NKH is higher in boys than in girls in China. Others may argue that this variation results from biased and/or intensive treatment during the early stages of illness in male infants compared to females.

Intractable seizures, lethargy, poor feeding, hypotonia, and unexplained apnea are the most common clinical manifestation in patients with NKH in China. Individuals with severe NKH exhibit intractable seizures and developmental stagnation, while those with the attenuated form display mild delays (expressive language impairment) and hyperactivity. All deceased cases exhibited abnormalities in brain MRI and/or EEG. Some literature suggests that typical EEG patterns, such as hypsarrhythmia and burst suppression, as well as severe cerebral malformations and dysplasia of the corpus callosum, often indicate a poor outcome, which is consistent with the results of this research (25).

**Figure 2:**
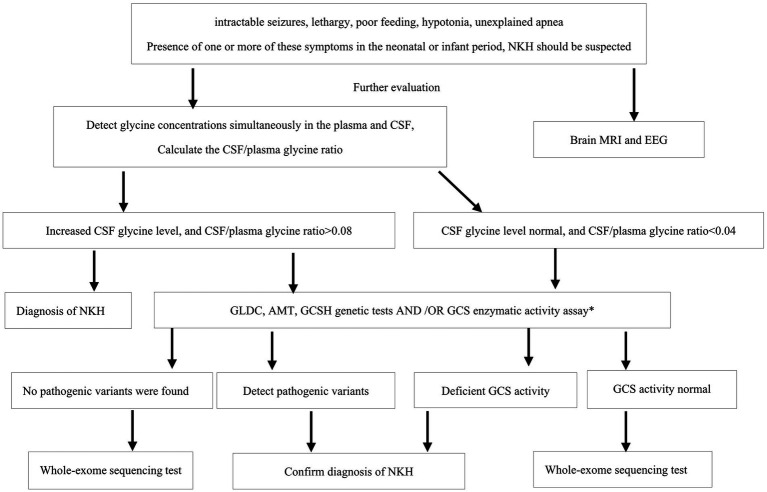
The NKH diagnostic flowchart. NKH, nonketotic hyperglycinemia; CSF, Cerebrospinal fluid; EEG, EIectroencephaIography; MRI, magnetic resonance imaging; GCS, glycine cleavage system. *GCS activity assay required a liver biopsy, as GCS activity is not expresses in fibroblasts nor untransformed lymphocytes.

An increased CSF glycine level, along with an elevated glycine index (CSF/plasma glycine ratio > 0.08), suggests the diagnosis of NKH (25). At present, NKH is often confirm diagnosed by genetic testing or enzymatic activity assay. And it is important to note that there is well-documented that normal glycine levels in serum and CSF do not definitively rule out NKH. While earlier research has indicated that neonatal-onset individuals have a poor prognosis compared to those with infantile-onset, and individuals with high elevated CSF to plasma glycine ratio tend to have a poorer prognosis. In our investigation, CSF glycine levels and glycine index help diagnose NKH, but they may not predict disease severity accurately, as indicated by the favorable outcomes of patient 17. This finding aligns with the conclusions drawn by Boneh et al. (26). Based on our research, we propose a diagnostic process for NKH in China, as depicted in Figure 2.

Mutations in *GLDC* and *AMT* genes exhibit high heterogeneity, with no consistent genotype–phenotype correlations reported. Clinical variability among siblings and different gene alteration prevalences compared to other populations suggest regional genetic diversity. In our cohort, we observed clinical variability in siblings (case 3, case 4) with the same genotype. While the reported prevalence of *GLDC/AMT* gene alterations in the literature is around 75 and 20%, respectively, our study observed a prevalence of 65 and 35%, aligning with findings from Turkish populations (27) and hinting at potentially higher AMT gene alteration rates in China.

Mutations in the *GLDC* gene can occur in various regions, including promoter regions, introns, and as copy number variations (CNVs). CNVs, which commonly result in a severe form of the condition, are suggested to be influenced by the genomic architecture of GLDC, including the presence of Alu elements (1, 28). A prevent missense mutation, p.R515S, observed in European patients was notably absent in Chinese patients, and no hotspot mutations were detected in the Chinese population (29). The mutation p.A802V in the *GLDC* gene has been linked to an attenuated form of the disease (30), as seen in Case 17. The novel mutation p.D880G, located in the carboxy-terminal part, has shown a higher pathogenic mutation rate (1).

Missense mutations are the most frequent mutation type in AMT. Consistent with the previous findings, recurrent missense mutations such as p.R222C, p. D276H, and p.R331Q were more prevalent in *AMT*, collectively accounting for 43.7% of all disease alleles. No intragenic CNVs were detected in this cohort.

Our report introduces novel heterozygous mutations in the *AMT* gene (p.R261G, p.H42P, and c.550 + 4A > G) and the *GLDC* gene (p.D880G), previously unreported in the Human Gene Mutation Database and were identified as shown in Table 3. Further research is needed to fully understand how these novel mutations contribute to pathogenicity and the resulting dysfunction in proteins for NKH.

**Table 3:** *In silico* analysis of novel mutations in our patients with NKH.

patient no.	Variant/*In silico* program	MutationTaster	FATHMM	SIFT	PROVEAN
16	GLDC gene c.2639A > G	Disease causing	Damaging −2.26	Damaging 0.000	Deleterious −6.5
17	AMT gene c.781A > G	Disease causing	Tolerated −1.19	Damaging 0.000	Deleterious −6.86
19	AMT gene c.125A > C	Disease causing	Damaging −1.94	Damaging 0.001	Deleterious −8.70

DNA mutation numbering is based on cDNA sequence: +1 corresponds to the A of the ATG translation initiation codon (GLDC: NM_000170.2; AMT: NM_000481.3).

Currently, no known cure or specific treatment exists for this devastating disease, and formal treatment guidelines are yet to be established. Current approaches involve using sodium benzoate to lower glycine levels in the bloodstream and N-methyl-D-aspartate receptor (NMDA) site antagonists such as dextromethorphan and ketamine to mitigate overstimulation of NMDA receptors. Treatment data from 16 patients in this cohort show that the majority of severe form patients received sodium benzoate (250~500 mg/kg/day) and/or dextromethorphan (5~15 mg/kg/day) therapy. Despite early administration of these treatments and achieving a temporary reduction in glycine plasma concentration, the seizures of some patients shown partial improvement, but this does not seem to significantly impact the ultimate outcome of the patients. Managing seizures in NKH poses a significant challenge, especially for patients with a severe form who often require multiple antiepileptic drugs. The case at our center demonstrated that administering luminal may effectively control seizures in neonates with NKH.

The limitation of this study is that the incidence was calculated based on a reporting system, which might be underestimated. In numerous instances, the absence of plasma and/or cerebrospinal fluid glycine data in our cases led to a less precise analysis. The literature (31) indicates the presence of abnormal prenatal findings in NKH. The diagnosis typically occurs postnatally, resulting in limited fetal stage data, with our dataset comprising only one case (case 19) with such information.

This study highlights the clinical differences and outcome of the patients based on mutation types in China. And we have introduced a diagnostic process for NKH (Figure 2). We believe that this proposed process will play a significant role in standardizing the diagnosis of NKH within China, potentially leading to more consistent and accurate identification of the condition.

This study also defines four novel heterozygous variations in *GLDC* and *AMT* genes associated with varying degrees of clinical severity and outcomes. The diverse mutations documented across various ethnic populations may offer insights into the genetic profiles unique to different nationalities and elucidate the clinical variability of the disease.

Moving forward, we aim to conduct further investigations into the correlation between the clinical phenotype and genotype of NKH, as well as the treatment strategies for the condition.

In conclusion, genetic sequencing aids in early detection of NKH cases, revealing varied outcomes. Intractable seizures, lethargy, poor feeding, hypotonia, unexplained apnea serve as key clinical indicators for the presentation of NKH. The initial clinical presentations, CSF glycine levels, and glycine index are helpful in diagnosing NKH. However, they may not accurately predict disease severity. MRI and EEG abnormalities, on the other hand, may indicate a poor prognosis. Crucially, identification of the disease can facilitate early genetic counseling and prenatal diagnosis for future pregnancies.

## Data Availability

The URL of the online repository/database used in this research is as follows: https://databases.lovd.nl/shared/individuals?search_created_by=04738. The ID is as follows: 00453011;00453012;00453013;00453014.
